# Universal HIV and Birth Cohort HCV Screening in San Diego Emergency Departments

**DOI:** 10.1038/s41598-019-51128-6

**Published:** 2019-10-09

**Authors:** Martin Hoenigl, Kushagra Mathur, Jill Blumenthal, Jesse Brennan, Miriam Zuazo, Melanie McCauley, Lucy E. Horton, Gabriel A. Wagner, Sharon L. Reed, Gary M. Vilke, Christopher J. Coyne, Susan J. Little

**Affiliations:** 1 Department of Medicine, Division of Infectious Diseases and Global Public Health, University of California San Diego, San Diego, California United States; 2 University of San Diego School of Medicine, San Diego, California United States; 3 Department of Emergency Medicine, University of California San Diego, San Diego, California United States; 4 Department of Pathology, University of California San Diego, San Diego, California United States

**Keywords:** HIV infections, Health services

## Abstract

Universal HIV and HCV screening in emergency departments (ED) can reach populations who are less likely to get tested otherwise. The objective of this analysis was to evaluate universal opt-out HIV and HCV screening in two EDs in San Diego. HIV screening for persons aged 13–64 years (excluding persons known to be HIV+ or reporting HIV testing within last 12 months) was implemented using a 4^th^ generation HIV antigen/antibody assay; HCV screening was offered to persons born between 1945 and 1965. Over a period of 16 months, 12,575 individuals were tested for HIV, resulting in 33 (0.26%) new HIV diagnoses, of whom 30 (90%) were successfully linked to care. Universal screening also identified 74 out-of-care for >12-months HIV+ individuals of whom 50 (68%) were successfully relinked to care. Over a one-month period, HCV antibody tests were conducted in 905 individuals with a seropositivity rate of 9.9% (90/905); 61 seropositives who were newly identified or never treated for HCV had HCV RNA testing, of which 31 (51%) resulted positive (3.4% of all participants, including 18 newly identified RNA positives representing 2% of all participants), and 13/31 individuals (42%) were linked to care. The rate of newly diagnosed HCV infections exceeded the rate of newly diagnosed HIV infections by >7-fold, underlining the importance of HCV screening in EDs.

## Introduction

Nearly 40,000 individuals in the United States were newly diagnosed with HIV in 2017^1^. Additionally, at the end of 2015, about 165,000 individuals in the United States were unaware of their HIV positive status, with particularly high rates observed among younger Latino or Black men with heterosexual risk^1^. Those currently unaware of their HIV infection are not only at risk of suffering personal health consequences but may also unknowingly transmit HIV to others. About 40% of new HIV infections are transmitted by individuals living with undiagnosed HIV^1^. As a result, the Centers of Disease Control and Prevention (CDC) currently recommends routine universal HIV screening for all persons 13–64 years of age^2^. Although targeted testing of those engaged in transmission risk behaviors, such as sexually active men who have sex with men (MSM) and people who inject drugs results in a higher yield of HIV diagnoses^3–6^, universal (i.e. opt-out) HIV screening in emergency department (ED) settings has the potential to reach populations who do not consider themselves at risk or are otherwise less likely to seek out and participate in HIV testing^7,8^. Another important aspect of universal HIV testing programs in the ED is finding known HIV+ individuals infected who have fallen out of care.

The ED setting has been demonstrated to be effective not only for HIV screening, but also for screening for hepatitis C virus (HCV) infection^9^. While the number of deaths associated with HCV had been increasing dramatically until 2013^10^, the introduction of direct-acting antiviral therapy has since turned HCV infection into a curable disease^11,12^. In response, a growing number of EDs are screening for HCV infection, particularly among baby boomers born between 1945 and 1965^13^. The CDC recommends routine HCV screening for this birth cohort, due to the high frequency of illicit drug use and contaminated transfusions that occurred in the 1970s and 80s^14^.

The objectives of this analysis were to evaluate universal opt-out HIV and birth-cohort HCV screening and determine the number of known HIV-positive individuals out of care identified through the program at two academic emergency departments in San Diego, California.

## Results

**Figure 1:**
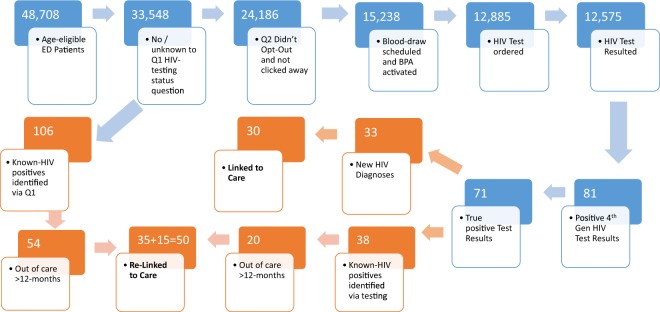
Flow diagram of testing algorithm (blue) and Case Management flow (brown). New HIV positives were identified only via testing, while known HIV positives were identified via testing and also question 1 of the automated EMR algorithm.

**Figure 2:**
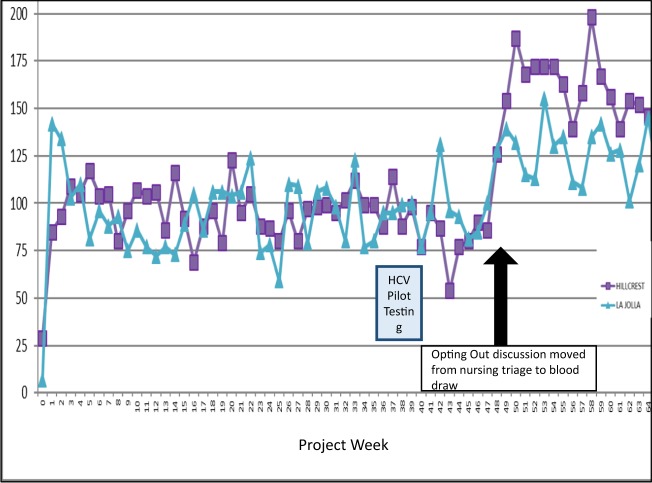
Number of HIV tests per week (number of tests Y-axis, week on X-axis) in the two participating Emergency Departments. Blue rectangle indicates the time period the HCV pilot was conducted. Arrow indicates the time when the opting-out discussion was moved from the nursing triage to the blood draw.

Between July 2017 and October 2018, 48,708 patients between 13 and 64 years of age visited the EDs, of which 33,548 (73%) answered “No/Unknown” to the question whether they had an HIV test during the last months or were HIV positive. Of those 33,548, 24,186 (72%) of patients did not opt out of HIV testing, and 15,238 (63%) of those had at least one visit with a blooddraw resulting in an automatic EMR order of the HIV test, which was signed by the provider and gave a HIV test result in 12,575 (83%) of individuals between 13 and 64 years of age, in whom a total of 14,759 HIV tests had been ordered (Flow diagram in Fig. 1). HIV testing increased significantly once the opt-out discussion was moved from the nursing triage process to the bedside at the time of the blood draw, which resulted in a drop in the opt-out rate from 37% to about 5% (Fig. 2, see also Methods section). Of 12,575 individuals, 81 (0.64%) had a confirmed positive (i.e. tested positive twice) result with the 4^th^ generation HIV antibody (Ab)/p24 antigen (Ag) test (Architect, Abbott, United States). Of those who tested positive, 10 (12.3%) were found to be false positives (i.e. confirmatory HIV testing resulted negative), and 38 had previously tested positive after review (EMR search, or self-report during the HIV disclosure call with the dedicated Infectious Diseases physician), resulting in 33 new HIV diagnoses (0.26% of individuals tested), including one case of acute HIV (Ab-negative, Ag-positive). Demographics of individuals newly diagnosed with HIV infection are displayed in Table 1. The Architect assay was found to have a per test specificity of 99.93%, and - after exclusion of those who had tested positive before - a positive test had a positive predictive value of 76.7%. Of the 33 individuals with new HIV diagnoses, 30 (90%) were successfully linked to care.

**Table 1:** Demographic characteristics of individuals with: newly diagnosed HIV infection, out-of-care known HIV positives, newly diagnosed with HCV infection, and known HCV RNA positives who were never treated for HCV.

Demographics	New HIV Diagnoses (33)	Out-of-care >12 months Known HIV positives (74)	New HCV Diagnoses (18)	Known HCV RNA positives never treated (13)
Age (median, IQR)	37 (28–48)	41 (32–51)	62 (60–64)	61 (55–63)
Male	24 (73%)	62 (84%)	14 (78%)	10 (77%)
Female	8 (24%)	12 (16%)	4 (22%)	3 (23%)
Transfemale	1 (3%)	3 (4%)	0	0
White	15 (45%)	33(45%)	10 (56%)	8 (62%)
Black	7 (21%)	20 (27%)	5 (28%)	2 (15%)
Native Hawaiian/Pacific Islander	0	2 (3%)	0	0
Asian	1 (3%)	0	0	1 (8%)
Bi-racial	10 (30%)	19 (26%)	3 (17%)	2 (15%)
Hispanic Ethnicity	13 (39%)	23 (27%)	3 (17%)	2 (15%)
Men who have sex with men	18 (55%)	40 (54%)	NA	NA
Heterosexual contact	12 (36%)	19 (26%)	NA	NA
Injection Drug Used	1 (3%)	3 (4%)	NA	NA
Other/unknown	2 (6%)	12 (16%)	NA	NA
HCV/HIV co-infection	0	5 (7%)	0	0

In total, 74 known HIV positive individuals out-of-care for >12 months were identified, including 5 who were found to be HCV co-infected (i.e. HCV diagnosis in EMR; Table 1). Demographics and risk factors of known HIV+ out of care individuals were similar to those newly diagnosed with HIV (Table 1). Overall, 50 (68%) of known HIV+ out of care for >12 months patients were successfully relinked to care.

During the 1-month pilot of HCV screening, 970 HCV antibody tests were performed on 905 persons born between 1945 and 1965 (i.e., baby boomer birth cohort). A total of 90 (9.9%) individuals tested positive by HCV-Ab, and 61/90 (68%) HCV-Ab positive persons who were newly identified or never treated for HCV had an HCV-RNA test ordered. Thirty-one of 61 (51%) tested positive for HCV RNA (3.4% of all participants including 18 newly identified HCV RNA positives representing 2% of all participants, Table 1), and 13/31 (42%) were successfully linked to care.

The results of HIV and HCV screening programs broken down by the two participating EDs are shown in Table 2. The rate of newly diagnosed HCV infections exceeded the rate of newly diagnosed HIV infections by >7-fold.

**Table 2:** Yield of HIV and HCV screening programs broken down by the two participating Emergency Departments (EDs).

	Hillcrest ED	La Jolla ED
# HIV Tests Conducted	7702 (52%)	7057 (48%)
New HIV Diagnoses	29 (88%)	4 (12%)
Known HIV positives >12 months out of care identified	71 (96%)	3 (4%)
# HCV Tests Conducted	489 (50%)	481 (50%)
New HCV Diagnoses	15 (83%)	3 (17%)
Known HCV RNA positives never treated	11 (85%)	2 (15%)

## Discussion

We successfully instituted universal opt-out HIV and a pilot birth-cohort-targeted HCV screening in two EDs in San Diego. Three major findings are evident. First, only 2.6/1000 individuals tested in the ED for HIV were newly diagnosed with HIV, which was lower than expected and may be explained by the high density of locally accessible HIV testing available in San Diego. Of those who were newly diagnosed with HIV infection, 90% were linked to care. Second, identification of HIV+ out of care individuals was more than two-times as common as new HIV diagnoses, and more than two-thirds were successfully relinked to care. Third, the rate of newly diagnosed HCV infections exceeded the rate of newly diagnosed HIV infections by >7-fold outlining the importance of screening for HCV in the ED.

Opt-out HIV ED screening programs have been shown to be feasible and accepted by the majority of patients^15^. Additionally, these programs have been shown to yield four times higher absolute numbers of new diagnoses than a physician-directed testing approach^16^. The yield of ED opt-out HIV testing programs varies substantially among settings. While an average of 0.4% of new HIV diagnoses were reported in nine United States EDs^9^, a rate of 0.26% was found here for the San Diego EDs. The diagnostic yield of ED op-out HIV screening programs may be negatively correlated to the magnitude of HIV testing uptake in the EDs catchment area: more testing uptake over time reduces not only the proportion of positives unaware of their HIV infection, but may also reduce HIV incidence^17^.

In San Diego, several free-of-charge community-based HIV screening programs have been available since 1996, and the largest program, the Early Test, provides around 4,000 tests annually and is located in close proximity to one of the Hillcrest EDs^4^. A previous study found that the Early Test has contributed to the observed decrease in new HIV diagnoses in San Diego^18^. Thus, HIV screening programs in San Diego specifically have been shown to be successful in both decreasing HIV diagnoses in the area, and terminating transmission chains where the program was marketed^4,6,15^. The low rate of 0.26% new HIV diagnoses that we observed in our ED HIV screening program may therefore be explained by the high density of free-of-charge HIV screening programs, and potentially also by increasing use of HIV pre-exposure prophylaxis in high risk individuals^19–21^.

Importantly, our program still yielded 33 new HIV diagnoses of which 90% were successfully linked to care, highlighting the importance of ED HIV screening in further tackling the HIV epidemic in San Diego. The demographics of new HIV diagnoses identified through our ED screening program differed slightly from those reported for new HIV diagnoses by the County of San Diego, with a higher proportion of new HIV diagnoses among African-American populations compared to the San Diego County average (13%)^22^. Compared to the Early Test community-based HIV screening program, our ED screening programs identified higher proportions of women, African-Americans, and individuals indicating heterosexual contact as their main risk factor^5,23^. Both community-based HIV screening programs and routine testing in EDs may therefore have a combined effect on reducing HIV transmission and infection in the community. The rate of 0.26% new HIV diagnoses found in this analysis is also lower than the rate of 0.43% new HIV diagnoses found in a hospital opt-out HIV screening program conducted in San Diego in 2009^24^, which may be attributed to the effectiveness of HIV-screening programs in San Diego.

In the context of HIV screening, dedicated case management time allowed for a high linkage to care rate (90%), suggesting that linkage to care protocols may improve by including the involvement of multidisciplinary non-ED professionals^25^. Additionally, prompt notification and post-test counseling of persons with positive test results facilitated rapid linkage to care.

Opt-out HIV screening programs in the ED may also be used to identify known HIV-positive out-of-care individuals who now account for 61.3% of HIV transmissions in the United States^26^. Relinking those out-of-care HIV positives into care is therefore another important measure that may help to curb the epidemic. Important factors associated with individuals falling out of care include moving to another area, changing providers, and incarceration^27^. Of the 74 out-of-care individuals identified in our screening program, more than two-thirds were relinked to care. Rates of relinkage with the help of a case manager or linkage specialist have been demonstrated elsewhere to be between 40 and 50%^27^. This rate is substantially lower than the 90% linkage-to-care rate for individuals with new HIV diagnoses. The lower percentage of successful relinkage may be due to the fact that the out-of-care population may have a higher proportion of individuals with which, for a variety of reasons, it is more difficult to maintain contact.

Our EMR algorithm included automation of eligibility criteria and auto-population of lab order forms. This allowed for a reduction in human error, larger scale testing, and easier tracking of testing outcomes. An EMR configuration modified for the purpose of opt-out testing has been shown to maximize number of patients tested as well as minimize missed testing opportunities^28^. Additionally, shifting the HIV testing opt-out discussion from the time of ED triage to the time of the blood draw resulted in a notable increase in HIV tests conducted. This phenomenon has been shown in other studies, particularly in EDs in Illinois^15^. Having the discussion at the time of the blood draw may be more efficient for providers and may appear more reasonable for patients who recognize that they are already slated for a blood draw. However, this protocol has the limitation of only testing patients scheduled for a blood draw.

In our study 4^th^ generation HIV Ab/p24 Ag testing (Architect, Abbott, United States) was highly specific, with the observed specificity of 99.93%, which was higher than the specificity of 99.7% previously reported for Architect testing^6^. Nevertheless, 23.3% of positive Architect results in individuals who were not known positives, were false positives, reflecting the low pretest probability in our cohort.

In the United States, an estimated 2.4 million individuals are living with HCV, with approximately 1 case per 100,000 reported in 2016^29^. Based on the rate of newly diagnosed HCV infections exceeding the rate of newly diagnosed HIV infections by >7 fold, it is clearly important to screen for HCV in San Diego EDs. These findings diverged from another HIV and HCV screening study completed in Oakland EDs which demonstrated higher rates of newly diagnosed HCV infections than HIV infections by only three-fold^9^. Possible explanations include a greater prevalence of HCV infection in San Diego. Our pilot screening program identified that 31 (3.4%) of those screened individuals had positive HCV RNA test results, similar to other HCV screening programs such as a large ED program in New Jersey^30^. Similar to other HCV screening programs focusing on the baby boomer population^31^, HCV testing in our ED setting also benefited from a robust EMR algorithm.

Challenges that need to be overcome when implementing ED HIV and HCV screening may include limited acceptability by providers, due to certain barriers including inadequate time, lack of resources, and concerns regarding follow-up care availability^32^. Additionally, providers may view routine HIV testing to be an unnecessary cost. However, while untargeted HIV screening has been shown to cost roughly $10,000 per additional infection identified^33^, this is still markedly below the calculated threshold for cost-savings per new HIV diagnosis derived by Farnham and colleagues^34^.

There are several limitations of our analysis. HIV testing numbers were not consistent over the course of the screening program, and were low particularly early after program initiation. Given that HCV screening was performed only during a one month pilot and only in the birth cohort, limits our ability to compare rates of new HIV and HCV diagnoses. Further, our conclusions may be limited to populations of San Diego County and thus may not be extrapolated to other populations. Additionally, HCV RNA testing was only performed in 60% of HCV Ab positive individuals, because reflex HCV RNA testing was not automated and had to be ordered manually. Also, no data on viral suppression were available for those successfully linked to care. Finally, a dedicated full time case manager was working on linking HIV and HCV positive individuals to care, and our linkage and re-linkage rates may therefore not be generalizable for EDs that do not have funding for care management resources.

In conclusion, more than 12,000 individuals were screened for HIV in our San Diego EDs, and 0.26% of those were newly diagnosed with HIV. The rate of newly diagnosed HCV infections exceeded the rate of newly diagnosed HIV infections by >7-fold, highlighting the importance of HCV screening in the ED.

## Material and Methods

Electronic medical record (EMR) based universal opt-out HIV screening was performed between July 2017 and September 2018. The one-month pilot EMR-based birth-cohort HCV screening program was initiated in March 2018. Both screening programs were conducted in parallel at two academic EDs in San Diego, California. The study locations were an urban based residency training ED and a suburban tertiary referral hospital ED with combined annual census of approximately 80,000 visits. Both EDs utilize EPIC as the EMR for patient care and documentation.

Opt-out HIV testing was conducted using an EMR-based screening algorithm for all persons aged 13–64 years. The eligibility of each patient was determined via EMR directed questions during the nursing triage process. Based on the responses, individuals were excluded from testing via questioning in those who: 1) were HIV-infected based on self-report or documentation in the EMR; 2) had been tested for HIV in the previous year based on self-report or documentation of test in the EMR (taken effect mid-October 2018); 3) lacked decisional capacity; or 4) were unable to make decisions due to language or other barriers. Additionally, patients were excluded from testing if they chose to opt out. Once eligibility was confirmed during the nursing triage process, a flag with a pre-populated HIV testing order appeared in the EMR when the physician ordered blood work. In June 2018, the algorithm was modified to reduce the number of questions during the nursing triage process to a single question: “Has the patient been tested for HIV during the last 12 months or is the patient known to be HIV positive?” For all patients with a “No/Unknown” response for whom automatic screening of the hospital EMR system did not find a HIV test result within the last year or HIV diagnosis, an automatic prepopulated test order popped up in the EMR if blood work was ordered, which had to be signed off by the provider. A discussion regarding the opt out option was moved from the triage process to the patient’s bedside during the blood draw procedure (i.e. nurse informed the patients “included in the blood work is a screening test for HIV. This is a CDC recommendation and is performed on all adult patients unless they refuse”).

HIV testing was performed using 4th Generation HIV p24 Ag/HIV Ab combination screening (Architect, Abbott, United States), with positive test results being confirmed by a second Architect test^6^, before being sent to a referral laboratory for confirmatory Genius HIV-1/HIV-2 differentiation immunoassay and 3^rd^ generation chemiluminescence (CIA), and, if discordant results, HIV nucleic acid amplification testing^35^.

The EMR algorithm also identified known HIV-positive individuals out of care for greater than 12 months. For those with HIV diagnoses in the EMR or “known HIV positive” selected as primary response, the triage nurse was prompted to respond YES or NO to a question about whether the last HIV care visit was within the last 12 months. If they patient responded NO, a case manager would contact them in an attempt to bring them back into care.

Positive 4^th^ generation p24 Ag/HIVAb results were sent electronically via the EMR to (a) the Infectious Diseases physician on call for the program who disclosed HIV diagnosis (or decided to wait for confirmatory test results, depending on the patients history and clinic); (b) the ordering ED physician; and (c) the full-time (i.e. 40-hours per week) HIV case manager who had her office in the immediate vicinity of the Hillcrest ED and was able to visit patients in the ED as well as those who were admitted. Details of known HIV+ persons out of care were sent daily to the case manager. If the patient had already been discharged from the ED when the test resulted, the disclosure would be completed by phone. If the patient was admitted to the hospital, disclosure would be in person.

Linkage and relinkage to care, defined as being seen by an HIV specialty care provider, was performed by the HIV case manager after disclosure. The case manager assisted with navigating insurance, finding providers, and making appointments, with final confirmation by phone that the individual successfully linked to care.

Opt-out HCV testing was completed only for individuals in the birth cohort born between 1945 and 1965 and whose ED care included an order for a blood draw. Opt-out HCV screening was performed using an HCV Ab tests with confirmatory HCV RNA tests ordered manually for those with positive HCV Ab test results. If sufficient sample was not available for HCV RNA testing, a follow-up visit was needed. The case manager disclosed HCV diagnosis and assisted with linkage to care, defined as being seen in a hepatology clinic or other specialty clinic that provided HCV treatment.

The project was sponsored by the FOCUS Program, which is a public health initiative that enables partners to develop and share best practices in routine blood-borne virus screening, diagnosis, and linkage to care in accordance with screening guidelines from the CDC, the U.S. Preventive Services Task Force (USPSTF), and state and local public health departments. All the project methods were carried out in accordance with relevant guidelines and regulations. The evidence based practice analysis was approved by the UCSD institutional review board which waived informed consent of participants.

Statistical analysis was performed using SPSS, version 25 (SPSS Inc., Chicago, IL, USA).
